# Altered White Matter Structural Network in Frontal and Temporal Lobe Epilepsy: A Graph-Theoretical Study

**DOI:** 10.3389/fneur.2020.00561

**Published:** 2020-06-17

**Authors:** Huan Lin, Xi Leng, Chunhong Qin, Wensheng Wang, Chi Zhang, Shijun Qiu

**Affiliations:** ^1^Department of Radiology, Zhujiang Hospital of Southern Medical University, Guangzhou, China; ^2^Department of Radiology, First Affiliated Hospital of Guangzhou University of Chinese Medicine, Guangzhou, China; ^3^Department of Radiology, Guangdong 999 Brain Hospital, Guangzhou, China

**Keywords:** epilepsy, white matter, network, diffusion tensor imaging (DTI), graph-theoretic

## Abstract

Temporal lobe epilepsy (TLE) and frontal lobe epilepsy (FLE) are the largest subgroup of partial epilepsy, and focal cortical dysplasias (FCDs) are highly epileptogenic brain lesions and are a frequent cause for antiepileptic drug (AED)-resistant focal epilepsies that mostly occur in the temporal and frontal lobes. We performed a graph-theoretical study based on the diffusion tensor imaging (DTI) data of patients with FLE or TLE caused by FCDs or lesions with high suspicion of FCDs and evaluated their cognitive function by the Chinese version of the Montreal Cognitive Assessment-Basic (MoCA-BC). The construction of the white matter structural network and graph-theoretical analysis was performed by Pipeline for Analysing Brain Diffusion Images (PANDA) and Graph-theoretical Network Analysis (GRETNA). We used the nonparametric analysis of covariance to compare the differences in diffusion metrics, network attributes and nodal attributes among FLE, TLE, and healthy control (HC) groups and then performed *post hoc* pairwise comparisons. Nonparametric Spearman partial correlation analysis was performed to analyse the correlation of network attributes with the age of onset, duration of disease, and MoCA-BC scores in patients with FLE and TLE. The results showed that the white matter structural network in patients with FLE and TLE was impaired in a more extensive set of regions than the FCD location. The similarities in white matter alterations between FLE and TLE suggested that their epileptogenic network might affect the fronto-temporal white matter tracts and thalamo-occipital connections, which might be responsible for the overlapping cognitive deficits in FLE and TLE. The white matter impairments in patients with FLE were more severe than those in patients with TLE, which might be explained by more affected nodes in the areas of DMN in patients with FLE.

## Introduction

Temporal lobe epilepsy (TLE) and frontal lobe epilepsy (FLE) are the largest subgroup of partial epilepsy (1). Focal cortical dysplasias (FCDs) are localized malformations of cortical development that mostly occur in the temporal and frontal lobes (2–4). FCDs are highly epileptogenic brain lesions and are a frequent cause for antiepileptic drug (AED)-resistant focal epilepsies (5, 6). The epileptic discharges caused by FCD are often spatially correlated with FCD lesions.

Epilepsy is considered a disorder of neural networks (7). Neural networks involved in seizure generation, maintenance and spread of epileptic activity comprise cortico-subcortical circuits (8, 9). Subcortical structures, such as white matter, are involved in epileptic activities. As a result of seizures and other factors, patients with epilepsy often have cognitive dysfunction (6, 10). Recent studies have shown that there were overlaps in cognitive dysfunction between patients with FLE and those with TLE (11), but the underlying white matter alterations are not clear.

Based on the background above, we put forward the following questions: Are there any similarities and distinctions in white matter abnormalities between patients with FLE and those with TLE? Are the abnormalities related to the overlapping cognitive deficits in FLE and TLE? Therefore, we performed a graph-theoretical study based on the diffusion tensor imaging (DTI) data of patients with FLE or TLE caused by FCDs or lesions with high suspicion of FCDs and evaluated their cognitive function, with the aim of discovering the characteristics in the white matter structural network in focal epilepsy of the frontal and temporal lobes and exploring the correlation of white matter network alterations with cognitive dysfunction.

## Methods

### Participants

#### FLE Group/TLE Group

All cases were patients with AED-resistant epilepsy in the Epilepsy Centre of Guangdong 999 Brain Hospital.

Inclusion standards: (1) patients were aged 15–45 years old and right-handed; (2) both clinical symptoms and long-term video electrography (EEG) indicated that focal epilepsy originated from the frontal or temporal lobe; and (3) conventional MRI showed that FCD lesions were confined to the unilateral frontal or temporal lobe.

Exclusion standards: (1) no ictal or interictal discharge was detected during long-term video EEG examination; (2) the epileptogenic region indicated by the clinical symptoms and EEG was inconsistent with the FCD lesions shown on conventional MRI; (3) multifocal FCD lesions or more than one lobe of the brain were involved in the monofocal FCD lesion; (4) in addition to FCD, there were other intracranial lesions, history of brain trauma or surgery, neurologic and psychiatric illness, or systemic disease affecting the central nervous system.

#### Healthy Control (HC) Group

All controls were recruited from healthy volunteers.

Inclusion standards: aged 15–45 years old, roughly age-matched with the FLE/TLE patient group, and right-handed.

Exclusion standards: intracranial lesions or anatomical variations, history of brain trauma or surgery, neurologic and psychiatric illness, or systemic disease affecting the central nervous system.

### Clinical Data and Cognitive Function Assessment

#### Clinical Data

Before MR scanning, we recorded the name, sex, and date of birth of all subjects. For epilepsy patients, we recorded the age of onset, birth history, past history, and family history of epilepsy and recorded the clinical symptoms and long-term video EEG results in the medical record.

#### Cognitive Function

Cognitive function was evaluated by one researcher using the Chinese version of the Montreal Cognitive Assessment-Basic (MoCA-BC) (12, 13). The total score and module scores (executive function, fluency, orientation, calculation, abstraction, delayed recall, visual perception, naming, and attention) of each subject were recorded.

### Image Acquisition

The MR images were acquired by a Philips Gyroscan Intera 1.5T magnetic resonance scanner.

#### Conventional MRI

Patients with epilepsy underwent an epilepsy package scan (T1, T2, FLAIR) to diagnose and locate FCD and to exclude those with other intracranial lesions. If the FCD lesions were unclear on the package scan, a T2-FLAIR scan (3-mm slice thickness in the axial, coronal, and sagittal positions) was added. Healthy controls received a conventional T2-FLAIR sequence scan to exclude those with intracranial lesions and anatomical variations.

#### DTI and 3D-T1

All subjects underwent a DTI and 3D-T1 sequence scan. (1) Diffusion tensor single-shot plane echo sequence: TR, 11000 ms; TE, 74 ms; FOV, 210 ^*^ 210 mm; voxel size, 2 ^*^ 2 ^*^ 2 mm; 67 layers; 32 diffusion gradient directions, *b* = 800 s/mm. (2) Sagittal T1-weighted 3D gradient echo sequence: TR, 25 ms; TE, 4.6 ms; FOV, 230 ^*^ 230 mm; voxel size, 1 ^*^ 1 ^*^ 1 mm; 170 layers; layer gap, 0 mm; scanning angle 0°.

### MR Imaging Diagnosis of FCD

The MR epilepsy package scan and T2-FLAIR scan (3-mm slice thickness) images were blindly interpreted by at least two experienced neuroimaging radiologists. The diagnostic criteria for FCD were as follows: increased cortical thickness, blurring of the cortical-white matter junction, increased signal on T2 weighted images, a radially-oriented linear or conical transmantle stripe of T2 hyperintensity, cortical thinning, and localized brain atrophy (4). The left/right side and locations of the FCD lesions were recorded (Supplementary Material 1). For TLE patients, we also recorded whether they had the MRI findings of hippocampal sclerosis (HS), including marked atrophy on coronal T1-weighted images, hyperintensity on T2-weighted and FLAIR images, and loss of hippocampal internal architecture clarity (14). If the diagnoses were different, a consensus was reached through a discussion.

### Data Processing and Graph-Theoretical Analysis

Data processing and graph-theoretical analysis were performed in MATLAB R2012a (The MathWorks, Inc, US). We used the Pipeline for Analysing the Brain Diffusion Image (PANDA) (15) package for data pre-processing and white matter structural network construction and used the Graph-theoretical Network Analysis (GRETNA) (16) package to calculate the network attributes and nodal attributes.

#### Pre-processing

(1) Converted the DTI and 3D-T1 data from DICOM format to the 4D NIfTI format; (2) corrected the image distortion that was caused by the eddy current and head movement; (3) extracted the brain from the images to improve the accuracy of registration; (4) with the DTI data coregistered to the high-resolution 3D-T1 images, subsequently, the coregistered images were normalized into the Montreal Neurological Institute (MNI) 152 space; (5) based on the JHU white-matter tractography atlas, obtained the fractional anisotropy (FA), axial diffusivity (AD), radial diffusivity (RD) of each white matter region.

#### Construction of the White Matter Structural Network

(1) Network node definition: the entire brain was divided into 90 regions using Anatomical Automatic Labelling (AAL) 90 atlas, where each region represented a network node. (2) Deterministic fiber tracking was implemented with Diffusion Toolkit (http://trackvis.org/dtk/), using Fiber Assignment by Continuous Tracking (FACT) algorithm. Terminated fiber tracking if two consecutive moving directions had crossing angle above 45°, or if the FA was out of the threshold 0.2~1 (17). (3) Network construction was based on deterministic fiber tracking. Based on whole brain fibers, fiber number matrix was created.

#### Calculating the Network and Nodal Attributes

Since the network matrix was created based on DTI data, we selected “similarity threshold” as the threshold type, and the threshold represented the number of streamline connecting the nodes. However, no golden criteria was available for which streamline number was currently the most biologically meaningful. Referring to previous study (17), a threshold range of 0–5 with an interval of 1 was chosen. When the streamline number between the two nodes was greater than the threshold, it was considered that there was an actual fiber connection. The network attributes above each threshold were displayed in the line chart, and a representative threshold was selected for calculating the network attributes.

The small-world networks were characterized by a balance between global integration and local specification of information transfer in the complex brain network (17). The high cluster coefficient and high local efficiency would reflect the optimization of the local network, while the short shortest path length and high global efficiency would reflect the optimization of the global network. Sigma, also known as “small-worldness,” was the quantification of the balance of local and global network. The degree number of the node was an indicator for evaluating the importance of nodes in the network. In this study, the following network attributes were calculated: the cluster coefficient and gamma (the ratio of the cluster coefficient in this study and that of a random network), the shortest path length and lambda (the ratio of the shortest path length in this study and that of a random network), the sigma (the ratio of gamma and lambda), the local efficiency of the network, and the global efficiency of the network. The following nodal attributes were calculated: the degree number of the node and the local efficiency of the node.

### Statistical Analysis

We used the mean (standard deviation), median (interquartile range) or frequency (proportion) for statistical description. Normality tests were performed using the Shapiro–Wilk test.

The χ^2^ test was used to compare the sex distribution among the FLE, TLE, and HC groups. The Kruskal-Wallis H test was performed to compare the age distribution among the three groups. The independent-samples t test was used to analyse the differences in the age of onset between the FLE and TLE groups. The Mann-Whitney *U*-test was used to analyse the differences in the duration of disease between the two patient groups. The χ^2^ test was used to compare the location of FCD (left/right side) between the two patient groups.

The nonparametric analysis of covariance (18) was used to analyse the differences in the MoCA-BC scores, diffusion metrics, the network attributes and nodal attributes among the FLE, TLE, and HC groups (after controlling for age and sex), and the *post hoc* pairwise comparisons were performed. The nonparametric analysis of covariance was also used to analyse the difference in the network attributes and nodal attributes among the FCD I, II, III groups, and between the HS and non-HS groups (after controlling for age, sex, age of onset, and duration of disease). The nonparametric Spearman partial correlation was performed to analyse the correlation of network attributes with the age of onset (after controlling for age, sex and duration of disease), the correlation of network attributes with the duration of disease (after controlling for age, sex and age of onset), and the correlation of network attributes with MoCA-BC scores (after controlling for age, sex, age of onset, and duration of disease) in the FLE and TLE groups.

We used IBM SPSS 23.0 for all univariate analysis and the nonparametric Spearman partial correlation (Supplementary Material 2) and used “sm” package in R software for nonparametric analysis of covariance (Supplementary Material 3). The FDR method was applied to correct for multiple comparisons of the MoCA-BC scores, the diffusion metrics and the nodal attributes. The Bonferroni method was applied to correct for *post hoc* pairwise comparisons. We considered a two-tailed *P* value of < 0.10 to be statistically significant for the Shapiro–Wilk Test and a two-tailed *P* value of < 0.05 to be statistically significant for other testing.

## Results

### Baseline Characteristics (Supplementary Material 1)

There were no statistically significant differences in sex (χ^2^ = 2.18, *P* = 0.336) or age (χ^2^ = 5.36, *P* = 0.068) among the FLE, TLE, and HC groups; there were no statistically significant differences in age of onset (*t* = 1.95, *P* = 0.058), duration of disease (*Z* = 0.53, *P* = 0.466), or FCD location (χ^2^ = 0.00, *P* = 1.000) between the FLE and TLE groups (Table 1).

**Table 1:** Baseline characteristics of the FLE, TLE, and HC groups.

**Characteristics**	**FLE**	**TLE**	**HC**
*n*	22	22	22
Sex			
Male	16 (72.7%)	16 (72.7%)	12 (54.5%)
Female	6 (27.3%)	6 (27.3%)	10 (45.4%)
Age (years)	22.5 (19.0, 26.3)	23.5 (17.0, 33.3)	26.0 (25.0, 28.0)
Age of onset (years)	13.9 (5.5)	17.0 (5.1)	-
Duration of disease (years)	9.0 (3.8, 15.3)	5.0 (3.0, 14.5)	-
Location of FCD			
Left	12 (54.5%)	12 (54.5%)	-
Right	10 (45.5%)	10 (45.5%)	-

### Cognitive Function Assessment

The median (interquartile range) MoCA-BC total scores of the FLE, TLE and HC groups were 24.0 (21.3, 27.0), 23.5 (19.0, 27.0), and 28.5 (27.0, 29.0), respectively. The differences in the MoCA-BC total score and the fluency, orientation, abstraction and delayed recall scores were statistically significant among the three groups (adjusted *P* < 0.05) (Table 2).

**Table 2:** MoCA-BC total score and module scores of the FLE, TLE, and HC groups.

**Characteristics**	**Scores (median)**			**Adjusted** ***P*** values^*^			
	**FLE**	**TLE**	**HC**	**All groups**	**FLE vs. HC**	**TLE vs. HC**	**FLE vs. TLE**
Total score	24.0	23.5	28.5	**0.001**	**0.001**	**0.001**	1.000
Executive function	0.5	0.5	1.0	0.123	-	-	-
Fluency	1.0	1.0	2.0	**0.001**	**0.001**	**0.002**	1.000
Orientation	5.0	6.0	6.0	**0.003**	**0.003**	**0.003**	0.924
Calculation	3.0	3.0	3.0	0.314	-	-	-
Abstraction	2.0	2.0	3.0	**0.001**	**0.002**	**0.002**	0.297
Delayed recall	3.0	2.0	4.0	**0.003**	**0.003**	**0.005**	0.754
Visual perception	3.0	3.0	3.0	0.621	-	-	-
Naming	4.0	4.0	4.0	0.314	-	-	-
Attention	3.0	3.0	3.0	1.000	-	-	-

* *The FDR method was applied to correct for multiple comparisons of the MoCA-BC scores, and the Bonferroni method was applied to correct for post hoc pairwise comparisons. The bold face P values were statistically significant (adjusted P < 0.05)*.

### Diffusion Metrics

The white matter regions with altered FA values and RD values in FLE and TLE patients were shown in Table 3. There was no statistically significant difference in the AD values in all of the white matter regions among the three groups.

**Table 3:** The white matter regions with altered FA, RD values in FLE and TLE patients.

**White matter regions**	**Adjusted** ***P*** values^*^		
	**All groups**	**FLE vs. HC^a^**	**TLE vs. HC^b^**
**WHITE MATTER REGIONS WITH ALTERED FA VALUES**			
Left anterior thalamic radiation	**0.003**	**0.004**	0.085
Right anterior thalamic radiation	**0.007**	**0.002**	0.636
Left corticospinal tract	**0.007**	**0.005**	0.477
Left cingulum (hippocampus)	**0.002**	**0.016**	**0.001**
Forceps major	**0.004**	**0.002**	0.276
Forceps minor	**0.002**	**0.002**	0.195
Left inferior fronto-occipital fasciculus	**0.002**	**0.001**	**0.003**
Right inferior fronto-occipital fasciculus	**0.002**	**0.001**	**0.015**
Left inferior longitudinal fasciculus	**0.002**	**0.005**	**0.004**
Right inferior longitudinal fasciculus	**0.002**	**0.005**	**0.002**
Left uncinate fasciculus	**0.036**	**0.014**	0.067
Right uncinate fasciculus	**0.002**	**0.003**	0.156
**WHITE MATTER REGIONS WITH ALTERED RD VALUES**			
Left superior longitudinal fasciculus	**0.006**	**0.004**	**0.016**
Right superior longitudinal fasciculus	**0.006**	**0.007**	**0.017**

* *The FDR method was applied to correct for multiple comparisons of FA values in 20 white matter regions, and the Bonferroni method was applied to correct for post hoc pairwise comparisons. The bold face P values were statistically significant (adjusted P < 0.05)*.

a *The white matter regions with a significantly lower FA value or a significantly higher RD value in the FLE group than in the HC group*.

b *The white matter regions with a significantly lower FA value or a significantly higher RD value in the TLE group than in the HC group*.

*There was no statistically significant difference in the FA or RD values between the FLE and TLE groups*.

### Threshold Selection

**Figure 1:**
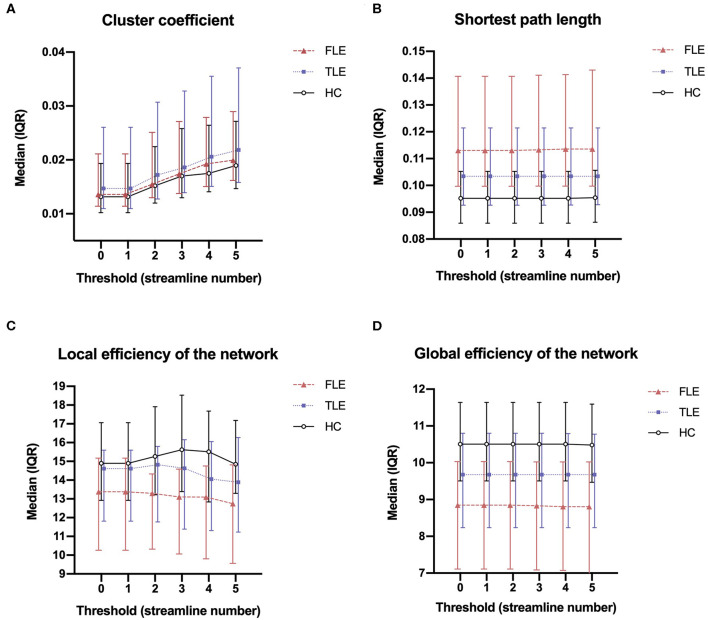
The median (interquartile range) of the cluster coefficient **(A)**, shortest path length **(B)**, local efficiency of the network **(C)**, and global efficiency of the network **(D)** of the three groups across thresholds of 0, 1, 2, 3, 4, and 5.

The cluster coefficient, the shortest path length, the local efficiency of the network, and the global efficiency of the network were calculated using thresholds of 0, 1, 2, 3, 4, and 5 (Figure 1). The four network attributes were relatively stable across different thresholds. The cluster coefficient was higher, and the shortest path length was longer in the FLE and TLE groups compared with the HC group, while the local efficiency and global efficiency of the network was lower. The alterations in the shortest path length, the local efficiency of the network, and the global efficiency of the network were more significant in the FLE group. We selected 3 as the threshold to calculate the network attribute values for the following statistical analysis (17).

### Network Attributes

#### Cluster Coefficients and Shortest Path Lengths

**Figure 2:**
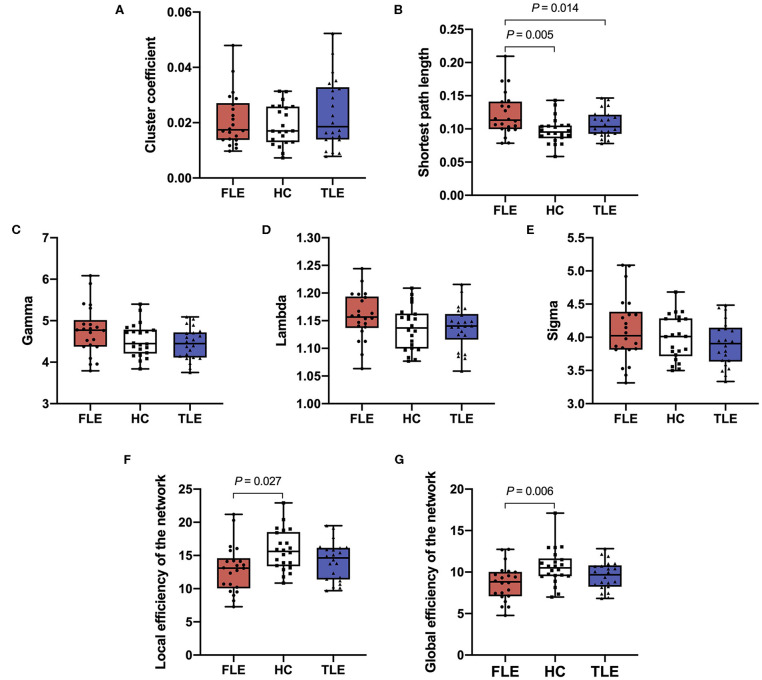
The cluster coefficient **(A)**, shortest path length **(B)**, gamma **(C)**, lambda **(D)**, sigma **(E)**, local efficiency of the network **(F)**, and global efficiency of the network **(G)** of the three groups.

There was no statistically significant difference in the cluster coefficients among the FLE, TLE, and HC groups (*P* = 0.292). There were statistically significant differences in the shortest path length among the three groups (*P* = 0.001). *Post hoc* pairwise comparisons showed that the shortest path length in the FLE group was significantly longer than that in the HC group (adjusted *P* = 0.005), and that in the TLE group (adjusted *P* = 0.014). There was no statistically significant difference between the TLE and HC groups (adjusted *P* = 0.458) (Figure 2).

#### Gamma, Lambda, and Sigma

There were statistically significant differences in gamma (*P* = 0.010) and sigma (*P* = 0.048) among the FLE, TLE, and HC groups, but there was no statistical significant difference in *post hoc* pairwise comparisons (adjusted *P* > 0.05). There was no statistical significance in lambda (*P* = 0.201) among the three groups (Figure 2).

#### Local Efficiency and Global Efficiency of the Network

There were statistically significant differences in the local efficiency (*P* = 0.019) and global efficiency (*P* = 0.004) among the three groups. *Post hoc* pairwise comparisons showed that the local efficiency and global efficiency in the FLE group were significantly lower than those of the HC group (adjusted *P* = 0.027, adjusted *P* = 0.006), and there were no statistically significant differences between the TLE and HC groups (adjusted *P* = 0.693, adjusted *P* = 0.447) or the FLE and TLE groups (adjusted *P* = 0.398, adjusted *P* = 0.263) (Figure 2).

### The Correlation of the Network Attributes With the Clinical Data and Cognitive Function

**Figure 3:**
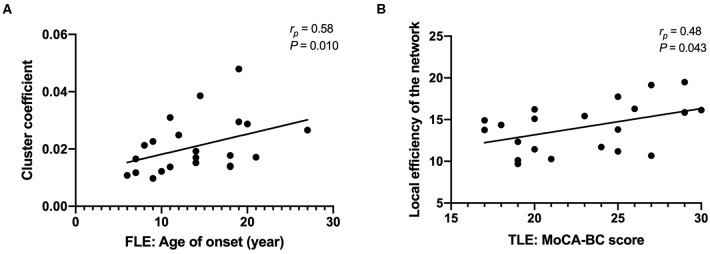
The cluster coefficients were positively correlated with the age of onset after controlling for age, sex, and duration of disease in the FLE group **(A)**; the local efficiency of the network was positively correlated with the MoCA-BC total score after controlling for age, sex, age of onset, and duration of disease in the TLE group **(B)**.

The cluster coefficient was positively correlated with the age of onset after controlling for age, sex, and duration of disease in the FLE group (*r*_*p*_ = 0.58, *P* = 0.010) (Figure 3). The local efficiency of the network was positively correlated with the MoCA-BC total score after controlling for age, sex, age of onset, and duration of disease in the TLE group (*r*_*p*_ = 0.48, *P* = 0.043) (Figure 3). There were no correlations between other network attributes (cluster coefficient, shortest path length, gamma, lambda, sigma, local efficiency of the network, global efficiency of the network) and the age of onset, the duration of disease and the MoCA-BC total scores.

### The Network Attributes in Patients With Different Type of FCD and Patients With/Without HS

Six patients with FLE underwent the resection of epileptic focus, 2 of which were FCD type I, and 4 of which were FCD type II. Two patients with TLE underwent the resection of epileptic focus, all of which were FCD type III (Supplemental Material 1). There was no statistically significant difference in the network attributes among the patients with FCD type I, II, and III (*P* > 0.05).

Of the 22 patients with TLE, 12 patients had imaging findings of HS (Supplemental Material 1). The shortest path length in patients with HS was significantly longer than that in patients without the imaging findings of HS (*P* = 0.002). The global efficiency of the network in patients with HS was significantly lower than that in patients without the imaging findings of HS (*P* = 0.030). There was no statistically significant difference in the cluster coefficient, gamma, lambda, sigma, local efficiency of the network between the TLE patients with HS and without HS (*P* > 0.05).

### Nodal Attributes

#### Degree Number of the Nodes

**Figure 4:**
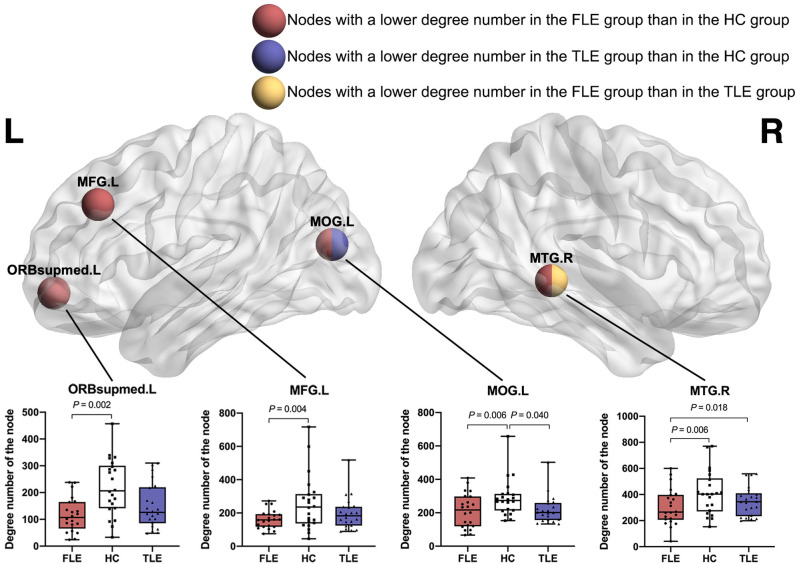
The nodes with a significantly lower degree number in the FLE group than in the HC group (red ball, adjusted *P* < 0.05), the nodes with a significantly lower degree number in the TLE group than in the HC group (blue ball, adjusted *P* < 0.05), and the nodes with a significantly lower degree number in the FLE group than in the TLE group (yellow ball, adjusted *P* < 0.05).

Compared with the HC group, the FLE group had a significantly lower degree number in the left middle frontal gyrus (MFG.L) (adjusted *P* = 0.004), left superior frontal gyri, medial orbital gyri (ORBsupmed.L) (adjusted *P* = 0.002), left middle occipital gyrus (MOG.L) (adjusted *P* = 0.006), right middle temporal gyrus (MTG.R) (adjusted *P* = 0.006). Compared with the HC group, the TLE group had a significantly lower degree number in the left middle occipital gyrus (MOG.L) (adjusted *P* = 0.040). Compared with the TLE group, the FLE group had a significantly lower local efficiency in the right middle temporal gyrus (MTG.R) (adjusted *P* = 0.018) (Figure 4).

#### Local Efficiency of the Nodes

There was no statistically significant difference in the local efficiency in all of the nodes among the FLE, TLE, and HC groups (*P* > 0.05).

## Discussion

### “Small-World Network” Characteristics in FLE and TLE Patients

In this study, we performed a graph-theoretical analysis based on the DTI data of FLE patients, TLE patients and healthy controls. Regarding network attributes, we compared their “small-world network” attributes and the local/global efficiency of the network. We found that the attributes of both the controls and the patients included a high cluster coefficient with low shortest path length and a high global efficiency with high local efficiency. The sigma values of all the three groups were greater than 1. This indicated that the white matter structural network in the controls and patients were all consistent with “small-world network” characteristics.

However, the shortest path length was longer, and the local/global efficiency was lower in the FLE patients than in the HCs. This revealed that the information transmissions between remote and regional brain regions were less effective in the FLE patients. The decreased local and global efficiency in patients with malformations of cortical development has also been reported in previous fMRI study (19). The longer shortest path lengths suggested that the global integration of the brain was decreased (20). These changes in the TLE patients had a similar trend but were not as significant as those in the FLE patients. This might indicate that the white matter impairments in the FLE patients were more severe than those in the TLE patients, and this might be related to differences in the characteristics of the white matter alteration between the two groups discussed below.

The cluster coefficients have been reported to be either increased or decreased in previous studies. Some researchers have observed that the cluster coefficients also changed during the progression of disease (20). We noticed that the cluster coefficients in the two patients groups were slightly higher than that in the HC group. Although the distinction was not statistically significant, we speculated that this trend might have indicated white matter reconstruction and abnormally increased local connectivity (19).

### White Matter Network Abnormalities Outside the Epileptogenic Zone

All patients with epilepsy included in this study had later pathologically confirmed FCDs or lesions with high suspicion of FCDs. The locations of the lesions were in accordance with the clinical symptoms and EEG results. These inclusion criteria maximized the likelihood that our patients had focal epilepsy originating from the frontal or temporal lobe, and the FCD location was equivalent to the epileptogenic zone. This made it reasonable to analyse the distribution of white matter alterations based on epilepsy from different origins.

In our study, the FLE and TLE group had widespread white matter abnormalities in integrity. The alteration of diffusion metrics were mainly manifested by the decrease of FA values and the increase of RD values, without AD abnormalities, which suggested widespread myelin degeneration, or maldevelopment (21). In addition, the nodes with decreased degree number in the FLE and TLE groups involved multiple lobes in the bilateral cerebral hemisphere. This revealed that the white matter structural network in FLE and TLE patients was impaired in a more extensive set of regions than the FCD location. FCDs were highly epileptogenic brain lesions and could widely affect the structural connectivity by a consequent net of axon degeneration (5, 21, 22). The wide-ranging connectivity changes might due to interictal/ictal spread of epileptic activity and the impact of seizures during brain development (11, 23).

### Similarities in White Matter Network Between FLE and TLE

There were similarities in white matter network alterations between FLE and TLE. This was not only reflected in the alteration of the diffusion metrics, as discussed above, but also in the alteration of the network and nodal attributes. In pairwise comparisons, there were no significant differences in the global efficiency and local efficiency of the network between the two patient groups. Regarding their nodal attributes, there were overlaps in the affected brain regions. Whether FLE or TLE, most of the affected brain regions were located in the frontal and temporal lobes. It was reported that children with FLE had smaller volumes of various cortex structures within the frontal lobes and in extra-frontal regions, most notably temporal cortex volumes (23). On the other hand, TLE has been reported to be associated with abnormal integrity of frontal-temporal white matter tracts (24). We speculated that the frontal-temporal connections were probably the vital parts of the epileptogenic network of both FLE and TLE.

In addition, we also found that the degree number of left middle occipital gyrus (MOG.L) decreased in both the FLE and TLE groups. Patients with TLE demonstrated abnormal thalamo-occipital functional connectivity (25). The thalamus played a critical role in epilepsy-generating mechanisms in focal seizures and exerted a major influence on the thalamocortical network and subcortical arousal systems (26). The thalamic arousal network dysfunction might contribute to the occipital gyrus alterations in patients of our study.

There were decreases in MoCA-BC total scores and several module scores in both the FLE and TLE groups, and there were overlaps in cognitive dysfunction between the two groups. The overlapping cognitive deficits in FLE and TLE might be explained by seizure activity that propagated from temporal to frontal areas (or vice versa) (11). The affected white matter network might be responsible for cognitive impairment, which would intensify over time (27).

### Distinctions in White Matter Network Between FLE and TLE

Compared with TLE, FLE group had decreased FA values in more white matter regions, including limbic, frontotemporal, temporooccipital, frontooccipital, and projection tracts. In addition, FLE had a greater influence on network attributes and had a wider range of influence on nodal attributes. It was worth noting that the patients with FLE had more affected nodes in the areas of DMN. The DMN was based on a set of brain areas that consistently activated in passive control situations, and could be associated with spontaneous cognition (21, 28). The alterations in DMN in patients with epilepsy has been reported in previous studies (21, 29, 30). In patients with FLE-FCD, decreased connectivity was detected in areas of the DMN ipsilateral to the epileptogenic zone, but this pattern of white matter disruption was not observed in the patients with TLE (21). The more global impairment of patients with FLE could be attributed to the long-lasting effects of frontal seizures (e.g., widespread inter- and intra-hemispheric propagation) (31).

The network attributes alterations in TLE with HS was more severe than those in the TLE without the imaging findings of HS (longer shortest path length and lower global efficiency of the network). Similar findings have also been reported in previous DTI and VBM analysis (21). The widespread white matter abnormalities would affect the structural connectivity of the hippocampus. However, whether HS was a consequence of altered structural connectivity, or a relatively independent process, needed further investigation.

### Limitations

This study had limitations worth discussion. The first limitation was that we didn't analyse the effects of AEDs on the white matter network. A higher number of AEDs might reflect greater seizure severity. Alternatively, it was possible that AEDs themselves might injure or impede developing white matter (32). The second limitation was that some patients did not undergo epileptogenic foci resection, so the lesions with high suspicion of FCDs were not confirmed by pathology. We will continue to follow up on the treatment and pathological results of our patients and expand the sample size. We hope that the characteristics of white matter network alterations caused by epileptogenic foci in different subregions of the frontal or temporal lobes can be explored in the future.

## Conclusion

The white matter structural network in FLE and TLE patients was impaired in a more extensive set of regions than in the FCD location. The similarities in nodal attributes alterations between FLE and TLE suggested that their epileptogenic network might affect the fronto-temporal white matter tracts and thalamo-occipital connections. These similarities might be responsible for the overlapping cognitive deficits in FLE and TLE. Compared with TLE, FLE had a greater influence on network attributes and had a wider range of influence on nodal attributes. This might be explained by more affected nodes in the areas of DMN in patients with FLE.

## Data Availability Statement

The datasets generated for this study are available on request to the corresponding author.

## Ethics Statement

The studies involving human participants were reviewed and approved by Guangdong 999 Brain Hospital. Written informed consent to participate in this study was provided by the participants' legal guardian/next of kin.

## Author Contributions

HL, SQ, and CZ designed this study. HL assessed the cognitive function. HL and WW organized the data. HL performed the data analysis and drafted the manuscript. XL, CQ, and SQ revised the manuscript. All authors approved the final manuscript.

## Conflict of Interest

The authors declare that the research was conducted in the absence of any commercial or financial relationships that could be construed as a potential conflict of interest.

## Abbreviations

FLE: frontal lobe epilepsy
TLE: temporal lobe epilepsy
FCD: focal cortical dysplasia
AED: antiepileptic drug
DTI: diffusion tensor image
MoCA-BC: the Chinese version of the Montreal Cognitive Assessment-Basic
PANDA: Pipeline for analysing brain diffusion images
GRETNA: Graph-theoretical Network Analysis
HC: healthy control
HS: hippocampal sclerosis
FA: fractional anisotropy
AD: axial diffusivity
RD: radial diffusivity
DMN: default mode network.
