# Supplementary material for: Altered White Matter Structural Network in Frontal and Temporal Lobe Epilepsy: A Graph-Theoretical Study
Source: Front Neurol. 2020 Jun 17;11:561. doi: 10.3389/fneur.2020.00561 (PMC7311567; doi:10.3389/fneur.2020.00561)
Supplement: Supplementary file 1 [file Data_Sheet_1.docx]

***Supplementary Material***

**1 Detailed baseline characteristics of the FLE and TLE patients**

| No. | group | sex | age | FCD location | | HS/  non-HS | surgical pathology |
| --- | --- | --- | --- | --- | --- | --- | --- |
|  |  |  |  | left/right | detailed location |  |  |
| 1 | FLE | male | 24 | left | paracentral lobule | - | - |
| 2 | FLE | female | 23 | left | inferior frontal gyrus | - | FCD IIB |
| 3 | FLE | male | 24 | left | anterior cingulate | - | - |
| 4 | FLE | male | 24 | left | precentral sulcus | - | FCD IIa |
| 5 | FLE | male | 22 | left | middle frontal gyrus | - | - |
| 6 | FLE | male | 28 | left | precentral gyrus | - | - |
| 7 | FLE | male | 27 | left | precentral gyrus | - | FCD IIB |
| 8 | FLE | male | 39 | left | orbital part of inferior frontal gyrus | - | - |
| 9 | FLE | female | 21 | left | anterior cingulate | - | - |
| 10 | FLE | male | 25 | left | supplementary motor area | - | - |
| 11 | FLE | female | 22 | left | superior frontal sulcus | - | FCD IIB |
| 12 | FLE | female | 17 | left | medial part of frontal lobe | - | - |
| 13 | FLE | male | 27 | right | inferior frontal gyrus | - | - |
| 14 | FLE | male | 19 | right | orbital part of frontal lobe | - | - |
| 15 | FLE | male | 27 | right | middle frontal gyrus | - | FCD IB |
| 16 | FLE | male | 18 | right | precentral gyrus | - | - |
| 17 | FLE | male | 22 | right | precentral gyrus | - | FCD I |
| 18 | FLE | female | 18 | right | anterior cingulate and genu of corpus callosum | - | - |
| 19 | FLE | female | 21 | right | precentral gyrus | - | - |
| 20 | FLE | male | 19 | right | superior frontal gyrus | - | - |
| 21 | FLE | male | 26 | right | inferior frontal gyrus | - | - |
| 22 | FLE | male | 19 | right | inferior frontal gyrus | - | - |
| 23 | TLE | male | 34 | left | medial temporal lobe | HS | - |
| 24 | TLE | male | 36 | left | superior temporal gyrus | non-HS | - |
| 25 | TLE | female | 45 | left | medial temporal lobe | HS | FCD IIIa |
| 26 | TLE | female | 17 | left | temporal pole and medial temporal lobe | HS | - |
| 27 | TLE | male | 22 | left | medial temporal lobe | HS | - |
| 28 | TLE | male | 23 | left | temporal pole | HS | - |
| 29 | TLE | female | 22 | left | middle temporal gyrus | non-HS | - |
| 30 | TLE | male | 17 | left | middle temporal gyrus | non-HS | - |
| 31 | TLE | male | 20 | left | medial temporal lobe | HS | - |
| 32 | TLE | male | 17 | left | temporal pole | non-HS | - |
| 33 | TLE | male | 36 | left | medial temporal lobe | HS | - |
| 34 | TLE | male | 28 | left | amygdala | non-HS | - |
| 35 | TLE | male | 21 | right | medial temporal lobe (including amygdala) | HS | - |
| 36 | TLE | male | 17 | right | inferior temporal gyrus | non-HS | - |
| 37 | TLE | female | 33 | right | amygdala | non-HS | - |
| 38 | TLE | male | 16 | right | superior temporal gyrus | non-HS | - |
| 39 | TLE | female | 24 | right | temporal pole | HS | - |
| 40 | TLE | male | 27 | right | anterior temporal lobe (collateral sulcus) | non-HS | - |
| 41 | TLE | female | 15 | right | medial temporal pole | HS | FCD IIIa |
| 42 | TLE | male | 31 | right | medial temporal lobe (including amygdala) | HS | - |
| 43 | TLE | male | 24 | right | medial temporal lobe (including amygdala) | non-HS | - |
| 44 | TLE | male | 35 | right | medial temporal lobe | HS | - |

**2 The R code for nonparametric analysis of covariance**

library(readxl)

library(sm)

library(writexl)

##read data, collate data, and view data##

degree<-read_excel("degree.xlsx",sheet=1)

degree$group<-factor(degree$group)

degree$sex<-factor(degree$sex)

str(degree)

eloc<-read_excel("eloc.xlsx",sheet=1)

eloc$group<-factor(eloc$group)

eloc$sex<-factor(eloc$sex)

str(eloc)

network<-read_excel("network.xlsx",sheet=1)

network$group<-factor(network$group)

network$sex<-factor(network$sex)

str(network)

FA<-read_excel("FA.xlsx",sheet=1)

FA$group<-factor(FA$group)

FA$sex<-factor(FA$sex)

str(FA)

AD<-read_excel("AD.xlsx",sheet=1)

AD$group<-factor(AD$group)

AD$sex<-factor(AD$sex)

str(AD)

RD<-read_excel("RD.xlsx",sheet=1)

RD$group<-factor(RD$group)

RD$sex<-factor(RD$sex)

str(RD)

## Perform nonparametric analysis of covariance to analyse overall differences among the FLE, TLE and HC groups for 90 brain regions in the data set named degree##

no<-c(1:90)

## Identify age and sex as covariates ##

cov<-c(degree$age,degree$sex)

## Create a dataframe to store the results of the analysis ##

pValue_degree<-data.frame(VAR_no=character(), pValue=numeric(), stringsAsFactors=FALSE)

for (i in no) {

result<-sm.ancova(x=cov, y=degree[,i+2],group=degree$group,model="equal")

pValue_degree[i,1]<-paste("VAR",i,sep = " ")

pValue_degree[i,2]<-result$p

}

pValue_degree<-pValue_degree[order(pValue_degree[,2]),]

adjusted_pValue<-p.adjust(pValue_degree$pValue,method="fdr",n=length(pValue_degree$VAR_no))

result_degree<-cbind(pValue_degree,adjusted_pValue)

## Write the results to an Excel file ##

write_xlsx(result_degree,path="result_degree(age&sex).xlsx",col_names=TRUE)

## Perform nonparametric analysis of covariance to analyse overall differences among the FLE, TLE and HC groups for 90 brain regions in the data set named eloc##

no<-c(1:90)

## Identify age and sex as covariates ##

cov<-c(eloc$age,eloc$sex)

## Create a dataframe to store the results of the analysis ##

pValue_eloc<-data.frame(VAR_no=character(), pValue=numeric(), stringsAsFactors=FALSE)

for (i in no) {

result<-sm.ancova(x=cov, y=eloc[,i+2],group=eloc$group,model="equal")

pValue_eloc[i,1]<-paste("VAR",i,sep = " ")

pValue_eloc[i,2]<-result$p

}

pValue_eloc<-pValue_eloc[order(pValue_eloc[,2]),]

adjusted_pValue<-p.adjust(pValue_eloc$pValue,method="fdr",n=length(pValue_eloc$VAR_no))

result_eloc<-cbind(pValue_eloc,adjusted_pValue)

## Write the results to an Excel file ##

write_xlsx(result_eloc,path="result_eloc(age&sex).xlsx",col_names=TRUE)

## Perform nonparametric analysis of covariance to analyse overall differences among the FLE, TLE and HC groups for 7 network attributes in the data set named network##

no<-c(3:9)

## Identify age and sex as covariates ##

cov<-c(network$age,network$sex)

## Create a dataframe to store the results of the analysis ##

pValue_network<-data.frame(VAR_no=character(), pValue=numeric(), stringsAsFactors=FALSE)

for (i in no) {

result<-sm.ancova(x=cov, y=network[,i],group=network$group,model="equal")

pValue_network[i,1]<-paste("VAR",i,sep = " ")

pValue_network[i,2]<-result$p

}

pValue_network<-pValue_network[order(pValue_network[,2]),]

## Write the results to an Excel file ##

write_xlsx(pValue_network,path="result_network(age&sex).xlsx",col_names=TRUE)

## Perform nonparametric analysis of covariance to analyse overall differences among the FLE, TLE and HC groups for 10 MoCA-BC scores in the data set named network##

no<-c(22:31)

## Identify age and sex as covariates ##

cov<-c(network$age,network$sex)

## Create a dataframe to store the results of the analysis ##

pValue_MoCA<-data.frame(VAR_no=character(), pValue=numeric(), stringsAsFactors=FALSE)

for (i in no) {

result<-sm.ancova(x=cov, y=network[,i],group=network$group,model="equal")

pValue_MoCA[i,1]<-paste("VAR",i,sep = " ")

pValue_MoCA[i,2]<-result$p

}

pValue_MoCA<-pValue_MoCA[order(pValue_MoCA[,2]),]

adjusted_pValue<-p.adjust(pValue_MoCA$pValue,method="fdr",n=length(pValue_MoCA$VAR_no))

result_MoCA<-cbind(pValue_MoCA,adjusted_pValue)

## Write the results to an Excel file ##

write_xlsx(result_MoCA,path="result_MoCA(age&sex).xlsx",col_names=TRUE)

## Perform nonparametric analysis of covariance to analyse overall differences among the FCD I, FCD II and FCD III groups for 7 network attributes in the data set named network##

no<-c(3:9)

## Identify age and sex as covariates ##

cov<-c(network$age,network$sex,network$onset,network$duration)

## Create a dataframe to store the results of the analysis ##

pValue_network<-data.frame(VAR_no=character(), pValue=numeric(), stringsAsFactors=FALSE)

for (i in no) {

result<-sm.ancova(x=cov, y=network[,i],group=network$FCD,model="equal")

pValue_network[i,1]<-paste("VAR",i,sep = " ")

pValue_network[i,2]<-result$p

}

pValue_network<-pValue_network[order(pValue_network[,2]),]

## Write the results to an Excel file ##

write_xlsx(pValue_network,path="result_network(age&sex)FCD.xlsx",col_names=TRUE)

## Perform nonparametric analysis of covariance to analyse overall differences between HS and non-HS groups for 7 network attributes in the data set named network##

no<-c(3:9)

## Identify age and sex as covariates ##

cov<-c(network$age,network$sex,network$onset,network$duration)

## Create a dataframe to store the results of the analysis ##

pValue_network<-data.frame(VAR_no=character(), pValue=numeric(), stringsAsFactors=FALSE)

for (i in no) {

result<-sm.ancova(x=cov, y=network[,i],group=network$HS,model="equal")

pValue_network[i,1]<-paste("VAR",i,sep = " ")

pValue_network[i,2]<-result$p

}

pValue_network<-pValue_network[order(pValue_network[,2]),]

## Write the results to an Excel file ##

write_xlsx(pValue_network,path="result_network(age&sex)HS.xlsx",col_names=TRUE)

## Perform nonparametric analysis of covariance to analyse overall differences among the FLE, TLE and HC groups for 20 white matter regions in the data set named FA##

no<-c(1:20)

## Identify age and sex as covariates ##

cov<-c(FA$age,FA$sex)

## Create a dataframe to store the results of the analysis ##

pValue_FA<-data.frame(VAR_no=character(), pValue=numeric(), stringsAsFactors=FALSE)

for (i in no) {

result<-sm.ancova(x=cov, y=FA[,i+2],group=FA$group,model="equal")

pValue_FA[i,1]<-paste("VAR",i,sep = " ")

pValue_FA[i,2]<-result$p

}

pValue_FA<-pValue_FA[order(pValue_FA[,2]),]

adjusted_pValue<-p.adjust(pValue_FA$pValue,method="fdr",n=length(pValue_FA$VAR_no))

result_FA<-cbind(pValue_FA,adjusted_pValue)

## Write the results to an Excel file ##

write_xlsx(result_FA,path="result_FA(age&sex).xlsx",col_names=TRUE))

## Perform nonparametric analysis of covariance to analyse overall differences among the FLE, TLE and HC groups for 20 white matter regions in the data set named AD##

no<-c(1:20)

## Identify age and sex as covariates ##

cov<-c(AD$age,AD$sex)

## Create a dataframe to store the results of the analysis ##

pValue_AD<-data.frame(VAR_no=character(), pValue=numeric(), stringsAsFactors=FALSE)

for (i in no) {

result<-sm.ancova(x=cov, y=AD[,i+2],group=AD$group,model="equal")

pValue_AD[i,1]<-paste("VAR",i,sep = " ")

pValue_AD[i,2]<-result$p

}

pValue_AD<-pValue_AD[order(pValue_AD[,2]),]

adjusted_pValue<-p.adjust(pValue_AD$pValue,method="fdr",n=length(pValue_AD$VAR_no))

result_AD<-cbind(pValue_AD,adjusted_pValue)

## Write the results to an Excel file ##

write_xlsx(result_AD,path="result_AD(age&sex).xlsx",col_names=TRUE)

## Perform nonparametric analysis of covariance to analyse overall differences among the FLE, TLE and HC groups for 20 white matter regions in the data set named RD##

no<-c(1:20)

## Identify age and sex as covariates ##

cov<-c(RD$age,RD$sex)

## Create a dataframe to store the results of the analysis ##

pValue_RD<-data.frame(VAR_no=character(), pValue=numeric(), stringsAsFactors=FALSE)

for (i in no) {

result<-sm.ancova(x=cov, y=RD[,i+2],group=RD$group,model="equal")

pValue_RD[i,1]<-paste("VAR",i,sep = " ")

pValue_RD[i,2]<-result$p

}

pValue_RD<-pValue_RD[order(pValue_RD[,2]),]

adjusted_pValue<-p.adjust(pValue_RD$pValue,method="fdr",n=length(pValue_RD$VAR_no))

result_RD<-cbind(pValue_RD,adjusted_pValue)

## Write the results to an Excel file ##

write_xlsx(result_RD,path="result_RD(age&sex).xlsx",col_names=TRUE)

##Perform post-hoc pairwise comparisons for indicators with statistically significant results in overall difference test, in the data set named degree##

no1<-c(1,2,3)

no2<-c(25,51,7,86)

pValue_degree<-data.frame(VAR_no=character(), pValue1_2=numeric(),pValue3_2=numeric(),pValue1_3=numeric(),stringsAsFactors=FALSE)

for (k in no1) {

if (k==1) {j=2}else if (k==2) {j=3}else if (k==3) {j=1}

data<-degree[which(degree$group== k | degree$group== j),]

cov<-c(data$age,data$sex)

for (i in no2) {

result<-sm.ancova(x=cov, y=data[,i+2],group=data$group,model="equal")

pValue_degree[i,1]<-paste("VAR",i,sep = " ")

pValue_degree[i,k+1]<-result$p

}

}

pValue_degree<-na.omit(pValue_degree)

##Perform Bonferroni correction for P values##

pValue_degree$pValue1_2<-pValue_degree$pValue1_2*3

pValue_degree$pValue3_2<-pValue_degree$pValue3_2*3

pValue_degree$pValue1_3<-pValue_degree$pValue1_3*3

## Write the results to an Excel file ##

write_xlsx(pValue_degree,path="pValue_degree for multiple comparisons(age&sex).xlsx",col_names=TRUE)

##Perform post-hoc pairwise comparisons for indicators with statistically significant results in overall difference test, in the data set named network##

no1<-c(1,2,3)

no2<-c(4,5,7,6,9)

pValue_network<-data.frame(VAR_no=character(), pValue1_2=numeric(),pValue3_2=numeric(),pValue1_3=numeric(),stringsAsFactors=FALSE)

for (k in no1) {

if (k==1) {j=2}else if (k==2) {j=3}else if (k==3) {j=1}

data<-network[which(network$group== k | network$group== j),]

cov<-c(data$age,data$sex)

for (i in no2) {

result<-sm.ancova(x=cov, y=data[,i],group=data$group,model="equal")

pValue_network[i,1]<-paste("VAR",i,sep = " ")

pValue_network[i,k+1]<-result$p

}

}

pValue_network<-na.omit(pValue_network)

##Perform Bonferroni correction for P values##

pValue_network$pValue1_2<-pValue_network$pValue1_2*3

pValue_network$pValue3_2<-pValue_network$pValue3_2*3

pValue_network$pValue1_3<-pValue_network$pValue1_3*3

## Write the results to an Excel file ##

write_xlsx(pValue_network,path="pValue_network for multiple comparisons(age&sex).xlsx",col_names=TRUE)

##Perform post-hoc pairwise comparisons for indicators with statistically significant results in overall difference test, in the data set named network##

no1<-c(1,2,3)

no2<-c(24,22,27,28,25)

pValue_MoCA<-data.frame(VAR_no=character(), pValue1_2=numeric(),pValue3_2=numeric(),pValue1_3=numeric(),stringsAsFactors=FALSE)

for (k in no1) {

if (k==1) {j=2}else if (k==2) {j=3}else if (k==3) {j=1}

data<-network[which(network$group== k | network$group== j),]

cov<-c(data$age,data$sex)

for (i in no2) {

result<-sm.ancova(x=cov, y=data[,i],group=data$group,model="equal")

pValue_MoCA[i,1]<-paste("VAR",i,sep = " ")

pValue_MoCA[i,k+1]<-result$p

}

}

pValue_MoCA<-na.omit(pValue_MoCA)

##Perform Bonferroni correction for P values##

pValue_MoCA$pValue1_2<-pValue_MoCA$pValue1_2*3

pValue_MoCA$pValue3_2<-pValue_MoCA$pValue3_2*3

pValue_MoCA$pValue1_3<-pValue_MoCA$pValue1_3*3

## Write the results to an Excel file ##

write_xlsx(pValue_MoCA,path="pValue_MoCA for multiple comparisons(age&sex).xlsx",col_names=TRUE)

##Perform post-hoc pairwise comparisons for indicators with statistically significant results in overall difference test, in the data set named FA##

no1<-c(1,2,3)

no2<-c(11,7,12,14,10,18,13,1,9,3,2,17)

pValue_FA<-data.frame(VAR_no=character(), pValue1_2=numeric(),pValue3_2=numeric(),pValue1_3=numeric(),stringsAsFactors=FALSE)

for (k in no1) {

if (k==1) {j=2}else if (k==2) {j=3}else if (k==3) {j=1}

data<-FA[which(FA$group== k | FA$group== j),]

cov<-c(data$age,data$sex)

for (i in no2) {

result<-sm.ancova(x=cov, y=data[,i+2],group=data$group,model="equal")

pValue_FA[i,1]<-paste("VAR",i,sep = " ")

pValue_FA[i,k+1]<-result$p

}

}

pValue_FA<-na.omit(pValue_FA)

##Perform Bonferroni correction for P values##

pValue_FA$pValue1_2<-pValue_FA$pValue1_2*3

pValue_FA$pValue3_2<-pValue_FA$pValue3_2*3

pValue_FA$pValue1_3<-pValue_FA$pValue1_3*3

## Write the results to an Excel file ##

write_xlsx(pValue_FA,path="pValue_FA for multiple comparisons(age&sex).xlsx",col_names=TRUE)

##Perform post-hoc pairwise comparisons for indicators with statistically significant results in overall difference test, in the data set named RD##

no1<-c(1,2,3)

no2<-c(16,15)

pValue_RD<-data.frame(VAR_no=character(), pValue1_2=numeric(),pValue3_2=numeric(),pValue1_3=numeric(),stringsAsFactors=FALSE)

for (k in no1) {

if (k==1) {j=2}else if (k==2) {j=3}else if (k==3) {j=1}

data<-RD[which(RD$group== k | RD$group== j),]

cov<-c(data$age,data$sex)

for (i in no2) {

result<-sm.ancova(x=cov, y=data[,i+2],group=data$group,model="equal")

pValue_RD[i,1]<-paste("VAR",i,sep = " ")

pValue_RD[i,k+1]<-result$p

}

}

pValue_RD<-na.omit(pValue_RD)

##Perform Bonferroni correction for P values##

pValue_RD$pValue1_2<-pValue_RD$pValue1_2*3

pValue_RD$pValue3_2<-pValue_RD$pValue3_2*3

pValue_RD$pValue1_3<-pValue_RD$pValue1_3*3

## Write the results to an Excel file ##

write_xlsx(pValue_RD,path="pValue_RD for multiple comparisons(age&sex).xlsx",col_names=TRUE)

**3 The SPSS syntax for nonparametric Spearman partial correlation analysis**

## Analyse the correlation of cluster coefficient with the age of onset (after controlling for age, sex and duration of disease) ##

define !psp (!pos !tokens(1)

/!pos !tokens(1)

/!pos !cmdend

/!pos !cmdend

/!pos !cmdend).

NONPAR CORR

/VARIABLES=!1 !2 !3 !4 !5

/PRINT=SPEARMAN TWOTAIL NOSIG

/MISSING=PAIRWISE

/matrix=out(*) .

RECODE ROWTYPE_ ('RHO'='CORR').

EXE.

PARTIAL CORR

/VARIABLES= !1 !2 BY !3 !4 !5

/SIGNIFICANCE=TWOTAIL

/FORMAT=MATRIX

/MISSING=LISTWISE

/MATRIX=IN(*).

!enddefine.

!psp Cp onset age sex duration..

## Analyse the correlation of shortest path length with the age of onset (after controlling for age, sex and duration of disease) ##

define !psp (!pos !tokens(1)

/!pos !tokens(1)

/!pos !cmdend

/!pos !cmdend

/!pos !cmdend).

NONPAR CORR

/VARIABLES=!1 !2 !3 !4 !5

/PRINT=SPEARMAN TWOTAIL NOSIG

/MISSING=PAIRWISE

/matrix=out(*) .

RECODE ROWTYPE_ ('RHO'='CORR').

EXE.

PARTIAL CORR

/VARIABLES= !1 !2 BY !3 !4 !5

/SIGNIFICANCE=TWOTAIL

/FORMAT=MATRIX

/MISSING=LISTWISE

/MATRIX=IN(*).

!enddefine.

!psp Lp onset age sex duration..

## Analyse the correlation of local efficiency of the network with the age of onset (after controlling for age, sex and duration of disease) ##

define !psp (!pos !tokens(1)

/!pos !tokens(1)

/!pos !cmdend

/!pos !cmdend

/!pos !cmdend).

NONPAR CORR

/VARIABLES=!1 !2 !3 !4 !5

/PRINT=SPEARMAN TWOTAIL NOSIG

/MISSING=PAIRWISE

/matrix=out(*) .

RECODE ROWTYPE_ ('RHO'='CORR').

EXE.

PARTIAL CORR

/VARIABLES= !1 !2 BY !3 !4 !5

/SIGNIFICANCE=TWOTAIL

/FORMAT=MATRIX

/MISSING=LISTWISE

/MATRIX=IN(*).

!enddefine.

!psp Eloc onset age sex duration..

## Analyse the correlation of global efficiency of the network with the age of onset (after controlling for age, sex and duration of disease) ##

define !psp (!pos !tokens(1)

/!pos !tokens(1)

/!pos !cmdend

/!pos !cmdend

/!pos !cmdend).

NONPAR CORR

/VARIABLES=!1 !2 !3 !4 !5

/PRINT=SPEARMAN TWOTAIL NOSIG

/MISSING=PAIRWISE

/matrix=out(*) .

RECODE ROWTYPE_ ('RHO'='CORR').

EXE.

PARTIAL CORR

/VARIABLES= !1 !2 BY !3 !4 !5

/SIGNIFICANCE=TWOTAIL

/FORMAT=MATRIX

/MISSING=LISTWISE

/MATRIX=IN(*).

!enddefine.

!psp Eg onset age sex duration..

## Analyse the correlation of cluster coefficient with the duration of disease (after controlling for age, sex and age of onset) ##

define !psp (!pos !tokens(1)

/!pos !tokens(1)

/!pos !cmdend

/!pos !cmdend

/!pos !cmdend).

NONPAR CORR

/VARIABLES=!1 !2 !3 !4 !5

/PRINT=SPEARMAN TWOTAIL NOSIG

/MISSING=PAIRWISE

/matrix=out(*) .

RECODE ROWTYPE_ ('RHO'='CORR').

EXE.

PARTIAL CORR

/VARIABLES= !1 !2 BY !3 !4 !5

/SIGNIFICANCE=TWOTAIL

/FORMAT=MATRIX

/MISSING=LISTWISE

/MATRIX=IN(*).

!enddefine.

!psp Cp duration age sex onset..

## Analyse the correlation of shortest path length with the duration of disease (after controlling for age, sex and age of onset) ##

define !psp (!pos !tokens(1)

/!pos !tokens(1)

/!pos !cmdend

/!pos !cmdend

/!pos !cmdend).

NONPAR CORR

/VARIABLES=!1 !2 !3 !4 !5

/PRINT=SPEARMAN TWOTAIL NOSIG

/MISSING=PAIRWISE

/matrix=out(*) .

RECODE ROWTYPE_ ('RHO'='CORR').

EXE.

PARTIAL CORR

/VARIABLES= !1 !2 BY !3 !4 !5

/SIGNIFICANCE=TWOTAIL

/FORMAT=MATRIX

/MISSING=LISTWISE

/MATRIX=IN(*).

!enddefine.

!psp Lp duration age sex onset..

## Analyse the correlation of global efficiency of the network with the duration of disease (after controlling for age, sex and age of onset) ##

define !psp (!pos !tokens(1)

/!pos !tokens(1)

/!pos !cmdend

/!pos !cmdend

/!pos !cmdend).

NONPAR CORR

/VARIABLES=!1 !2 !3 !4 !5

/PRINT=SPEARMAN TWOTAIL NOSIG

/MISSING=PAIRWISE

/matrix=out(*) .

RECODE ROWTYPE_ ('RHO'='CORR').

EXE.

PARTIAL CORR

/VARIABLES= !1 !2 BY !3 !4 !5

/SIGNIFICANCE=TWOTAIL

/FORMAT=MATRIX

/MISSING=LISTWISE

/MATRIX=IN(*).

!enddefine.

!psp Eg duration age sex onset..

## Analyse the correlation of local efficiency of the network with the duration of disease (after controlling for age, sex and age of onset) ##

define !psp (!pos !tokens(1)

/!pos !tokens(1)

/!pos !cmdend

/!pos !cmdend

/!pos !cmdend).

NONPAR CORR

/VARIABLES=!1 !2 !3 !4 !5

/PRINT=SPEARMAN TWOTAIL NOSIG

/MISSING=PAIRWISE

/matrix=out(*) .

RECODE ROWTYPE_ ('RHO'='CORR').

EXE.

PARTIAL CORR

/VARIABLES= !1 !2 BY !3 !4 !5

/SIGNIFICANCE=TWOTAIL

/FORMAT=MATRIX

/MISSING=LISTWISE

/MATRIX=IN(*).

!enddefine.

!psp Eloc duration age sex onset..

## Analyse the correlation of cluster coefficient with MoCA-BC scores (after controlling for age, sex, age of onset and duration of disease) ##

define !psp (!pos !tokens(1)

/!pos !tokens(1)

/!pos !cmdend

/!pos !cmdend

/!pos !cmdend).

NONPAR CORR

/VARIABLES=!1 !2 !3 !4 !5

/PRINT=SPEARMAN TWOTAIL NOSIG

/MISSING=PAIRWISE

/matrix=out(*) .

RECODE ROWTYPE_ ('RHO'='CORR').

EXE.

PARTIAL CORR

/VARIABLES= !1 !2 BY !3 !4 !5

/SIGNIFICANCE=TWOTAIL

/FORMAT=MATRIX

/MISSING=LISTWISE

/MATRIX=IN(*).

!enddefine.

!psp Cp MoCA age sex onset duration..

## Analyse the correlation of shortest path length with MoCA-BC scores (after controlling for age, sex, age of onset and duration of disease) ##

define !psp (!pos !tokens(1)

/!pos !tokens(1)

/!pos !cmdend

/!pos !cmdend

/!pos !cmdend).

NONPAR CORR

/VARIABLES=!1 !2 !3 !4 !5

/PRINT=SPEARMAN TWOTAIL NOSIG

/MISSING=PAIRWISE

/matrix=out(*) .

RECODE ROWTYPE_ ('RHO'='CORR').

EXE.

PARTIAL CORR

/VARIABLES= !1 !2 BY !3 !4 !5

/SIGNIFICANCE=TWOTAIL

/FORMAT=MATRIX

/MISSING=LISTWISE

/MATRIX=IN(*).

!enddefine.

!psp Lp MoCA age sex onset duration..

## Analyse the correlation of global efficiency of the network with MoCA-BC scores (after controlling for age, sex, age of onset and duration of disease) ##

define !psp (!pos !tokens(1)

/!pos !tokens(1)

/!pos !cmdend

/!pos !cmdend

/!pos !cmdend).

NONPAR CORR

/VARIABLES=!1 !2 !3 !4 !5

/PRINT=SPEARMAN TWOTAIL NOSIG

/MISSING=PAIRWISE

/matrix=out(*) .

RECODE ROWTYPE_ ('RHO'='CORR').

EXE.

PARTIAL CORR

/VARIABLES= !1 !2 BY !3 !4 !5

/SIGNIFICANCE=TWOTAIL

/FORMAT=MATRIX

/MISSING=LISTWISE

/MATRIX=IN(*).

!enddefine.

!psp Eg MoCA age sex onset duration..

## Analyse the correlation of local efficiency of the network with MoCA-BC scores (after controlling for age, sex, age of onset and duration of disease) ##

define !psp (!pos !tokens(1)

/!pos !tokens(1)

/!pos !cmdend

/!pos !cmdend

/!pos !cmdend).

NONPAR CORR

/VARIABLES=!1 !2 !3 !4 !5

/PRINT=SPEARMAN TWOTAIL NOSIG

/MISSING=PAIRWISE

/matrix=out(*) .

RECODE ROWTYPE_ ('RHO'='CORR').

EXE.

PARTIAL CORR

/VARIABLES= !1 !2 BY !3 !4 !5

/SIGNIFICANCE=TWOTAIL

/FORMAT=MATRIX

/MISSING=LISTWISE

/MATRIX=IN(*).

!enddefine.

!psp Eloc MoCA age sex onset duration..
